# Functional Ingredients in Minor Grain Crops

**DOI:** 10.3390/foods12061261

**Published:** 2023-03-16

**Authors:** Xiushi Yang, Cong Teng, Liang Zou, Guixing Ren

**Affiliations:** 1 Institute of Bast Fiber Crops, Chinese Academy of Agricultural Sciences, No. 348 Xianjiahu West Road, Changsha 410205, China; yangxiushi@caas.cn; 2 Institute of Crop Sciences, Chinese Academy of Agricultural Sciences, No. 12 Zhongguancun South Street, Haidian District, Beijing 100081, China; 3 Institute of Agro-Product Processing, Jiangsu Academy of Agricultural Sciences, No. 50 Zhongling Street, Xuanwu District, Nanjing 210018, China; 4 School of Food and Biological Engineering, Chengdu University, Chengdu 610106, China; 5 College of Life Science, Shanxi University, Taiyuan 030006, China

Minor grain crops are generally recognized as less-produced cereal or pseudo-cereal grain crops, excluding the four major grain crops of wheat, rice, corn, and soybean. The production of minor grain crops, including oat (*Avena sativa*), barley (*Hordeum vulgare*), foxtail millet (*Setaria italica*), buckwheat (*Fagopyrum esculentum*), sorghum (*Sorghum vulgare*), alday (*Coix lacryma-jobi*), and quinoa (*Chenopodium quinoa*), has been globally distributed and their cultivation area has gradually increased. In recent years, minor grain crops have gained much attention due to their functional ingredients, such as polyphenol, flavonoid, saponin, polysaccharide, and bioactive protein. These substances have considerable biological effects and health benefits. As important food materials, minor grains have been increasingly consumed, especially in developed areas with higher rates of chronic diseases. Therefore, it is meaningful to further identify the potential functional effects of minor grain crops and explore the main impact factors that could affect the content and bioactivity of their functional ingredients, such as processing methods, cooking methods, varieties, and environmental factors.

This Special Issue includes nine research articles containing multidisciplinary investigations of the functional ingredients of minor grain crops of coix seed, foxtail millet, buckwheat, quinoa, and sorghum. Coix seed, also called adlay, is a traditional Chinese medicine used in arthritis, diuretics, and pain relief. Yang et al. [1] found that the coix seed extract, mainly composed of coix seed oil, polysaccharide, and protein, could promote the growth, acidifying activity, and metabolism of *Limosilactobacillus reuteri*. This result indicated the prebiotic potential of coix seed. Furthermore, fermentation with *Bacillus subtilis* improved the anti-proliferative activity of dehulled adlay against six types of tumor cells, probably owing to the increase in functional ingredients, such as tetramethylpyrazine, γ-aminobutyric acid, and rutin [2].

Foxtail millet is an important crop that is widely planted in northern China and has long been used to cook porridge in everyday diets. Interestingly, the nutritional characteristics of millet porridge, including the fatty acid, protein, amino acid and microelement contents, could be affected by different electric cookers according to Zhang et al. [3]. This investigation might promote the further development of new electric cookers for cooking millet. While dehulling foxtail millet, a million tons of millet bran was produced. This by-product is rich in oil, dietary fiber and other nutrients, but has not been fully utilized. Recently, ethyl linoleate (ELA), a commercially valuable compound with health effects including enhancing immunity and reducing cholesterol and blood lipid levels, was identified in millet bran. In the research of Huang et al. [4], ELA was extracted and highly concentrated from millet bran oil via urea complexation and molecular distillation. This provides a new strategy for the extraction of ELA and shows the potential application of millet bran in health food. Furthermore, fermentation technology was utilized to increase the soluble dietary fiber content in millet bran by Chen et al. [5]. They found that the intestinal thrust rate and serum levels of immune factors were down-regulated, while the secretion of secretory immunoglobulin A in the intestinal mucosa was up-regulated by millet bran fermented with *Bacillus natto*. Their findings revealed the biological effect of fermented millet bran on relieving diarrhea induced by senna leaf in mice.

Tartary buckwheat is a pseudo-cereal that has been considered as a health food for the prevention and treatment of cardiovascular disease and diabetes owing to its inner flavonoids and protein. In the research of Liu et al. [6], the way in which Tartary buckwheat protein regulates lipid metabolism disorders was potentially attributed to the increase in the abundance of gut microbiota, as well as the improvement in short-chain fatty acids production and bile acid metabolism.

Quinoa is also a pseudo-cereal with comprehensive nutritional value, and the whole quinoa was considered to be helpful in the prevention of diabetes owing to its lower digestion rate. In the research of Dong et al. [7], heat-moisture treatments were successfully applied to quinoa flour to further lower its digestibility. The downregulation of digestibility and estimated glycemic index were due to the changes in the starch structure, such as the decrease in relative crystallinity and transformation of starch crystal.

Sorghum is an important crop for both the human diet and livestock fodder. The nutritional value and viscosity of sorghum were found to be affected by environmental factors including cultivation location and harvest year based on the study of 90 sorghum samples in northern Italy [8].

In addition to processing technology and environmental factors, the biotransformation method was also reported to affect the structure and biological activity of functional ingredients in this Special Issue. Hao et al. [9] found that O-methyltransferases could be used to transform liquiritigenin, naringenin, and hesperidin flavonoids. After bioreaction, five methylated flavonoids were obtained, and their antimicrobial and anti-breast cancer activities were further improved. This result indicated the potential of biotransformation technology for the sustainable production of functional ingredients.

Three review articles are also included in this Special Issue. Firstly, Ren et al. [10] summarized the recent research progress regfarding the use of functional ingredients in minor grain crops to alleviate chronic diseases, such as obesity, diabetes, cancer and cardiovascular diseases, by regulating gut microbiota. Furthermore, Qin et al. [11] clarified that fermentation could increase the content of functional ingredients, improve the digestibility of protein and carbohydrate, and decrease anti-nutritional factors including phytic acid and tannins in minor grains. Fermented minor grains are expected to be healthier than their counterparts to control blood glucose, blood lipids, and blood pressure levels. Finally, the research of Li et al. [12] focused on the bioactive compounds in sorghum, which is the fifth most cultivated cereal in the world. A series of traditional functional ingredients in sorghum, including phenolic compounds, carotenoids, vitamin E, amines, and phytosterols, were reviewed in this article, accompanied by their antioxidative, anticancer, antidiabetic, anti-inflammatory and anti-obesity properties. Additionally, the three newly detected isoflavones of formononetin, glycitein, and ononin were also mentioned.

In conclusion, the twelve papers collected in this issue cover a wide range of studies, from field trial research to processing technology investigation, with respect to the functional ingredients and healthy aspects of minor grain crops. These works are dedicated to enriching the knowledge of how functional ingredients in minor grain crops could benefit human health. We are very grateful to the authors, reviewers and staff of this journal for their scientific contribution and kind support of this issue. We sincerely hope that the readers will find this Special Issue informative and obtain the health benefits derived from consuming minor grain crops.
